# Neto2 Interacts with the Scaffolding Protein GRIP and Regulates Synaptic Abundance of Kainate Receptors

**DOI:** 10.1371/journal.pone.0051433

**Published:** 2012-12-06

**Authors:** Man Tang, Evgueni Ivakine, Vivek Mahadevan, Michael W. Salter, Roderick R. McInnes

**Affiliations:** 1 Program in Developmental and Stem Cell Biology, The Research Institute, Hospital for Sick Children, Toronto, Ontario, Canada; 2 Department of Molecular Genetics, University of Toronto, Toronto, Ontario, Canada; 3 Program in Neurosciences and Mental Health, The Research Institute, Hospital for Sick Children, Toronto, Ontario, Canada; 4 Department of Physiology, University of Toronto, Toronto, Ontario, Canada; 5 Lady Davis Research Institute, Jewish General Hospital, McGill University, Montreal, Quebec, Canada; University of Toronto, Canada

## Abstract

Kainate receptors (KARs) are a class of ionotropic glutamate receptors that are expressed throughout the central nervous system. The function and subcellular localization of KARs are tightly regulated by accessory proteins. We have previously identified the single-pass transmembrane proteins, Neto1 and Neto2, to be associated with native KARs. In the hippocampus, Neto1, but not Neto2, controls the abundance and modulates the kinetics of postsynaptic KARs. Here we evaluated whether Neto2 regulates synaptic KAR levels in the cerebellum where Neto1 expression is limited to the deep cerebellar nuclei. In the cerebellum, where Neto2 is present abundantly, we found a ∼40% decrease in GluK2-KARs at the postsynaptic density (PSD) of Neto2-null mice. No change, however, was observed in total level of GluK2-KARs, thereby suggesting a critical role of Neto2 on the synaptic localization of cerebellar KARs. The presence of a putative class II PDZ binding motif on Neto2 led us to also investigate whether it interacts with PDZ domain-containing proteins previously implicated in regulating synaptic abundance of KARs. We identified a PDZ-dependent interaction between Neto2 and the scaffolding protein GRIP. Furthermore, coexpression of Neto2 significantly increased the amount of GRIP associated with GluK2, suggesting that Neto2 may promote and/or stabilize GluK2:GRIP interactions. Our results demonstrate that Neto2, like Neto1, is an important auxiliary protein for modulating the synaptic levels of KARs. Moreover, we propose that the interactions of Neto1/2 with various scaffolding proteins is a critical mechanism by which KARs are stabilized at diverse synapses.

## Introduction

Kainate receptors (KARs) are functionally diverse ionotropic glutamate receptors with important roles in the regulation of synaptic transmission and neuronal excitability. On the presynaptic membrane, KARs regulate neurotransmitter release, and contribute to presynaptic forms of plasticity [1], [2]. At postsynaptic sites, some KARs function as ion channels that mediate a component of excitatory transmission [3], [4], [5], while others regulate neuronal excitability through a metabotropic action [6]. Given that the functions of KARs depend to a great extent on their localization, the targeting and stabilization of these receptors to the appropriate synapses and compartments must be a tightly regulated process. However, our current knowledge of the mechanisms behind these events and the regulatory elements involved is still rudimentary at best.

A limited number of KAR-associated molecules have been identified to date. Many of these are PDZ domain-containing proteins that bind directly to the receptors' cytoplasmic tail to control channel function, and/or subcellular localization. For example, the synaptically-enriched molecules PICK1 and GRIP have been implicated in stabilizing KARs on the postsynaptic membrane. Disruption of PDZ-dependent interactions, likely between KAR subunits and the PDZ domains of GRIP/PICK1, significantly reduced KAR-mediated synaptic transmission [7]. Neto1 and Neto2 are the only known transmembrane KAR auxiliary subunits. In heterologous expression systems, both Neto1 and Neto2 modulate receptor kinetics [8], [9], [10], and interact with recombinant KARs via their highly conserved extracellular CUB domains [11]. Furthermore, studies on native receptors have revealed that Neto1 controls not only the decay kinetics of KAR-mediated EPSCs [11], [12], but also the abundance of these receptors at the hippocampal postsynaptic membrane [11]. Unexpectedly, despite overlapping Neto2 and KAR expression in the hippocampus, the synaptic localization and function of these receptors were unchanged in Neto2-null mice [11]. Nonetheless, given the well-documented association of Neto2 with native KARs in the brain [8], [11], [12], and the increased synaptic accumulation of recombinant receptors in Neto2-transfected neurons [9], it is likely that, in certain synapses or brain regions, Neto2 participates in KAR regulation.

Here we examined whether Neto2 is involved in the synaptic localization of KARs in the cerebellum. In the cerebellar cortex, *Neto2* is robustly expressed in the internal granular layer and, to a lesser extent, Purkinje cells, while *Neto1* expression appears to be absent by *in situ* [13]. Additionally, given the important role of PDZ domain proteins in the organization, assembly, and localization of synaptic proteins [14], [15], we investigated whether Neto2, which has a putative class II PDZ binding motif, could interact with PDZ domain proteins functionally linked to KARs, and how such an interaction would affect their binding to these receptors.

## Results

### Synaptic abundance of KARs is reduced in the cerebellum of Neto2-null mice

**Figure 1:**
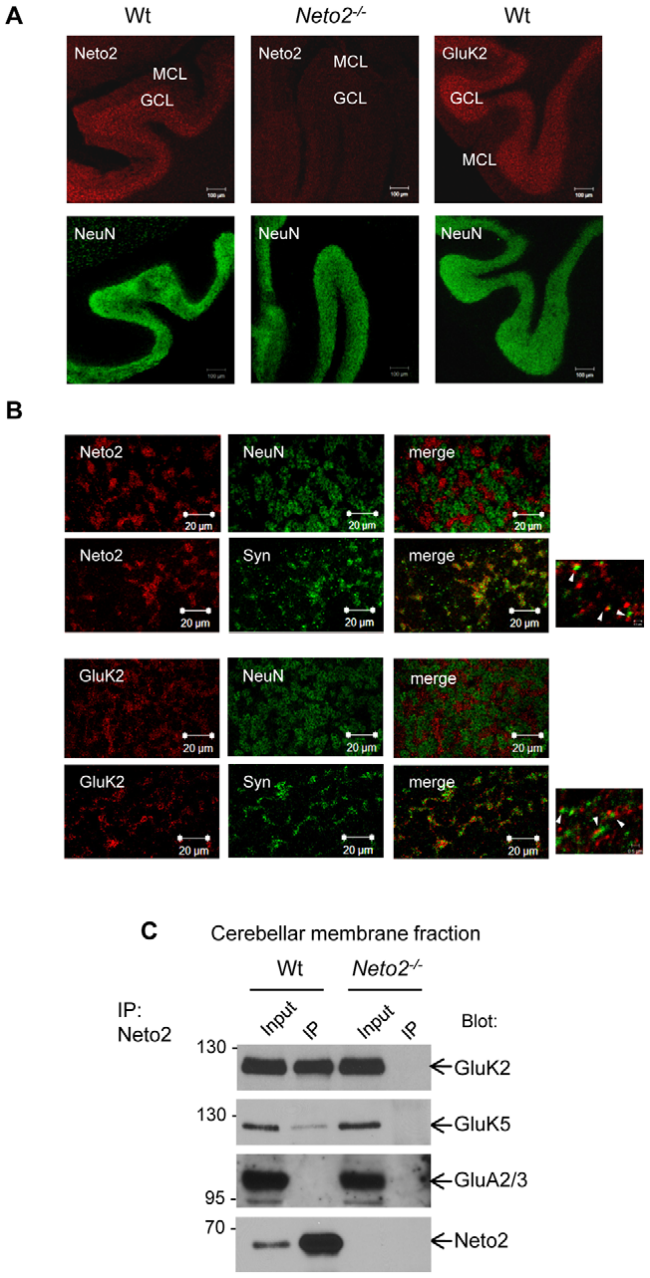
Neto2 is associated with KARs in the cerebellum. (A) Confocal micrographs of immunostained cerebellar slices. Antibodies used for immunostaining are indicated in the top left corner of each image. In the cerebellum, the NeuN antibody stains the neuronal nuclei of granule cells but does not recognize Purkinje cells. MCL, molecular cell layer; GCL, granule cell layer; Wt, wild-type sections; *Neto2^−/−^*, Neto2-null sections. Scale bar, 100 μm. (B) High-magnification confocal microscopy of the cerebellar granule cell layer immunostained with Neto2, GluK2, NeuN, or synaptophysin antibodies. Scale bar, 20 μm; scale bar (small panels on the right), 5 μm (C) Immunoblot of immunoprecipitates from the cerebellum. Blot: antibody used for immunoblot analysis; IP: immunoprecipitate. The input represents 2% of the material used in the immunoprecipitation experiment.

While KARs are prominently expressed in the cerebellar cortex [16], Neto1 appears to beabsent from this brain region [13]. Therefore, we examined the effect of Neto2, without interference of Neto1, on the synaptic expression of cerebellar KARs. To characterize the localization of the Neto2 protein, we performed immunofluorescent staining of cerebellar sections. As shown in Figure 1A, the granule cell layer (GCL), as detected by staining with the neuronal nuclear antigen, NeuN, exhibited the strongest Neto2 and GluK2 immunoreactivity, and displayed no obvious differences in thickness or cell density between wild-type and Neto2-null sections. Higher magnification images revealed that, within this layer, Neto2-, as well as, GluK2-positive structures had irregular shapes that were present in nuclear-free islets, which suggest an accumulation of these proteins in the cerebellar glomeruli (Figure 1B). To determine whether Neto2 and/or GluK2 were present at synapses, we double-immunostained the sections with synaptophysin. As seen in Figure 1B, Neto2, or GluK2 coclustered with synaptophysin in presumed GCL glomeruli. However, closer examination revealed that while the Neto2-, or GluK2-immunoreactive puncta was often found in close apposition to those from synaptophysin, they rarely showed complete overlap. This observation suggests that Neto2 and GluK2 may be present on postsynaptic sites associated with synaptophysin-positive axon terminals. No staining for Neto2 was observed in the Purkinje cell layer, which corresponds to the cell bodies of Purkinje cells, but a diffuse signal could be detected in the molecular layer (Figure 1A).

To determine whether Neto2 is associated with KARs in the cerebellum, we performed immunoprecipitation experiments from wild-type and Neto2-null cerebellar lysates. Anti-Neto2 antibodies coimmunoprecipitated the GluK2, and GluK5 KAR subunits from lysates of wild-type mice. However, neither GluK2, nor GluK5 were coimmunoprecipitated from Neto2-null samples (Figure 1C). Moreover, no coimmunoprecipitation was observed between Neto2 and the GluA2/3 subunit of the AMPARs (Figure 1C), indicating a specific interaction of KARs with Neto2.

**Figure 2:**
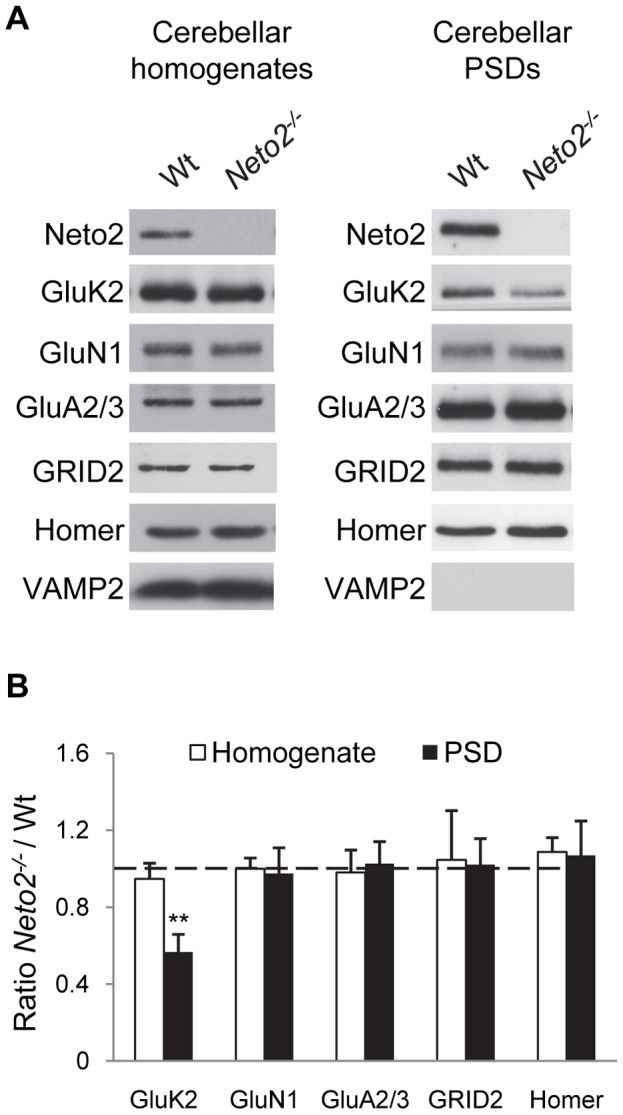
KARs are reduced in the cerebellar PSD of Neto2-null mice. (A) Immunoblots (representative of three experiments) of proteins from cerebellar homogenates and cerebellar PSD fractions of wild-type (Wt) and Neto2-null (*Neto2^−/−^*) mice. Antibodies used for detection are indicated on the left. (B) Histogram showing normalized levels of different proteins in Neto2-null cerebellar homogenates relative to that of wild-type (white bars), and in Neto2-null cerebellar PSD fractions relative to that of wild-type (black bars); **, p<0.01, paired t-test, n = 3.

To investigate whether Neto2 plays a role in the synaptic localization of KARs, we isolated cerebellar PSDs from wild-type and Neto2-null mice, and quantified relative protein levels by densitometry analysis of immunoblots. In Neto2-null PSDs, we observed a significant reduction of GluK2 KAR subunits when compared to wild-type mice (56%±9% of wild-type; mean ± SD, *p<0.01*) (Figure 2A, B), while the abundance of other synaptic proteins tested were all similar between the two genotypes (Figure 2A, B). To test whether the expression of Neto1 is activated in the absence of Neto2, we evaluated Neto1 protein levels in PSD samples by immunoblot analysis. We could not, however, detect Neto1 in cerebellar PSDs of either wild-type, nor Neto2-null samples (data not shown), suggesting that there is no compensation by Neto1 in Neto2-null mice, and that Neto1 is not responsible for the synaptic localization of the remaining KARs.

To determine whether the reduction of GluK2-KARs in the PSD was the result of changes in the amount of total protein, we compared GluK2 levels in wild-type and Neto2-null cerebellar homogenates. Immunoblot analysis of GluK2 and other cerebellar proteins showed that their abundance was no different between wild-type and Neto2-null mice (Figure 2A, B). Taken together, our results indicate that Neto2 is critical for the localization of KARs in PSDs in the cerebellum.

### Neto2 interacts with the PDZ domain scaffolding protein GRIP

**Figure 3:**
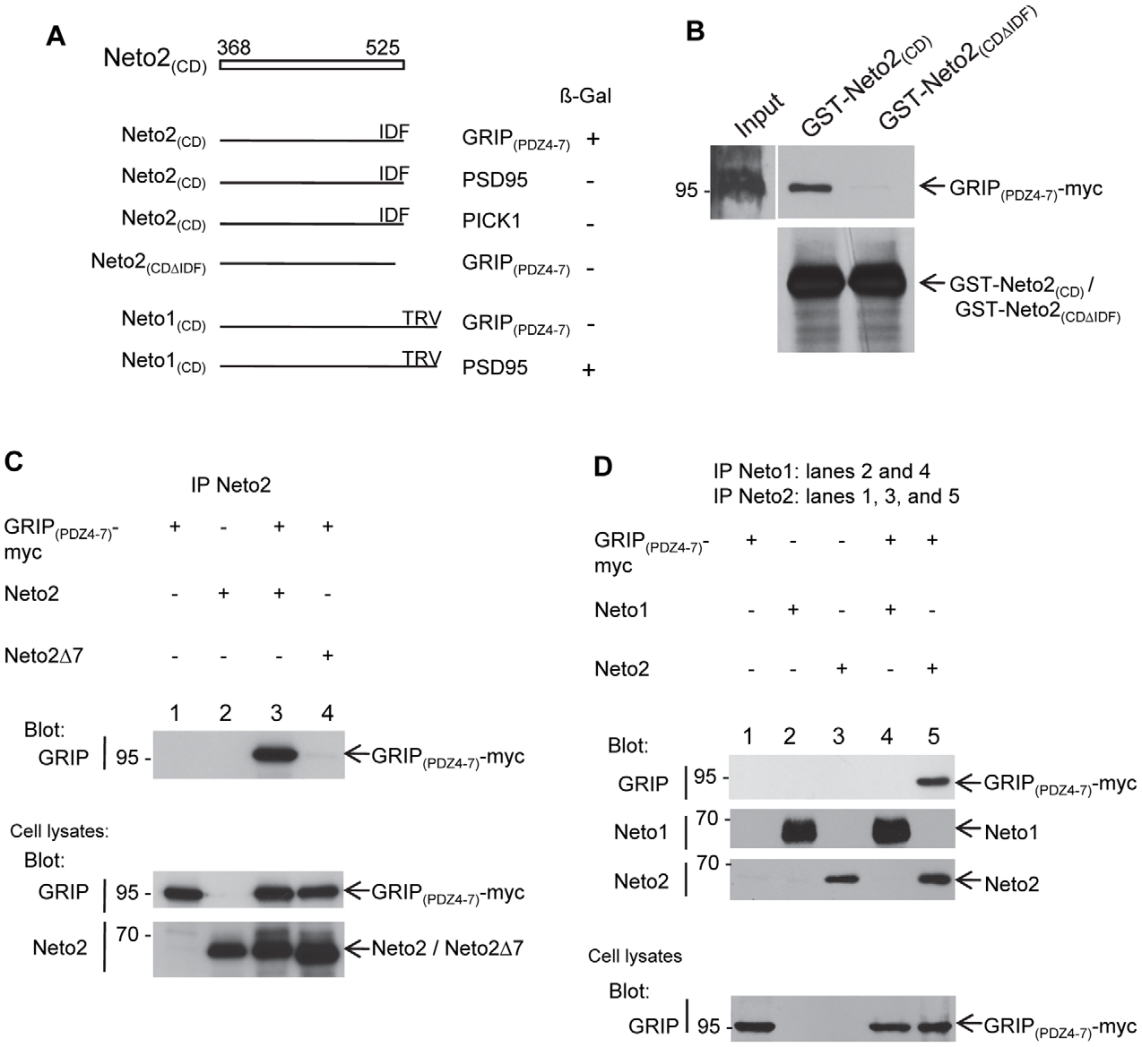
Neto2 interacts with GRIP through a C-terminal binding motif. (A) Yeast two-hybrid analysis of Neto2 interaction with PDZ domain-containing proteins. ß-gal, ß-galactosidase assay. Numbers on top of the white bar indicate the amino acid residues corresponding to the Neto2 cytoplasmic domain. Neto2_(CD)_, cytoplasmic domain of Neto2; Neto2_(CDΔIDF)_, cytoplasmic domain of Neto2 lacking the last three C-terminal amino acids (IDF); Neto1_(CD)_, cytoplasmic domain of Neto1 encompassing amino acids 345–533. The last three C-terminal amino acids of Neto1 are TRV. (B) Pull-down of recombinant GRIP_(PDZ4–7)_-myc with GST fused to the cytoplasmic domain of Neto2 (GST-Neto2_(CD)_) or with GST fused to the deletion mutant ΔIDF (GST-Neto2_(CDΔIDF)_). (C, D) Immunoblots of immunoprecipitates from transfected COS-7 cell lysates. The cDNAs used for transfection are shown above each lane. Neto2Δ7, Neto2 lacking the last seven C-terminal residues; blot, antibody used for immunoblot analysis; IP, antibody used for immunoprecipitation.

Given that Neto2 has a putative class II PDZ ligand at the C-terminus, we asked whether it may bind to PDZ domain proteins previously implicated in regulating the synaptic levels of KARs. We initially used the yeast-two hybrid system to test the interaction of Neto2 cytoplasmic domain (Neto2_(CD)_) with GRIP, and PICK1, two proteins that are known to bind to class II PDZ motifs, and have proposed roles in the stabilization of KARs at the postsynaptic membrane [7]. In two-hybrid assays, we found that coexpression of Neto2_(CD)_ with a fragment encoding PDZ 4–7 of GRIP (GRIP_(PDZ4–7)_) resulted in positive lacZ reporter gene activity based on ß-galactosidase assays, suggesting an interaction between the two molecules. On the other hand, we did not observe any interaction between Neto2_(CD)_ and the PDZ domain of PICK1 (Figure 3A). To determine whether the last three C-terminal residues of Neto2 mediate the interaction with GRIP_(PDZ4–7)_, we generated a Neto2_(CD)_ truncation mutant (Neto2_(CD_−_ΔIDF)_). Coexpression of Neto2_(CD_−_ΔIDF)_ and GRIP_(PDZ4–7)_ did not result in activation of the *lacZ* gene, suggesting that the deleted residues constitute a PDZ binding motif that is critical for the Neto2:GRIP_(PDZ4–7)_ interactions (Figure 3A). As a control, we also tested Neto2_(CD)_ against the class I PDZ domain protein PSD95. No interaction between these two proteins was observed, indicating a selective binding of Neto2 to GRIP. In contrast, Neto1_(CD)_, which has a class I binding motif, showed interaction with PSD95, in agreement with previously reported observations [17], but did not bind to GRIP_(PDZ4–7)_ (Figure 3A). Together, our yeast two-hybrid studies showed that GRIP might be a novel interacting partner for the intracellular domain of Neto2.

**Figure 4:**
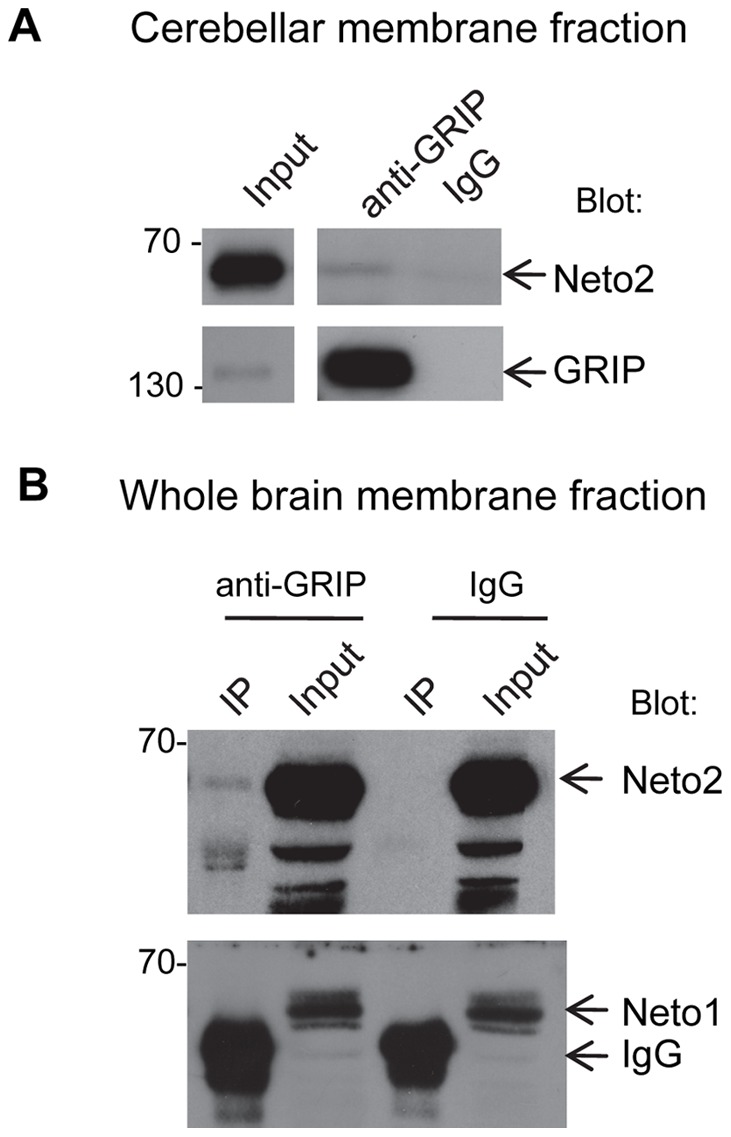
Neto2 associates with GRIP *in vivo*. (A, B) Immunoblots of immunoprecipitates from cerebellar (A) or whole brain (B) membrane fractions. Samples were subjected to immunoprecipitation with an anti-GRIP antibody, or with normal rabbit IgGs (IgG), as the negative control. Blot, antibody used for immunoblot analysis; IP, immunoprecipitate.

To determine whether the interaction between Neto2 and GRIP_(PDZ4–7)_ may be observed using an independent approach, we performed GST pull-down experiments, and coimmunoprecipitation with Neto2 and GRIP_(PDZ4–7)_ expressed in COS-7 cells. For GST pull-down, equal amounts of GST-Neto2_(CD)_, and GST-Neto2_(CDΔIDF)_ fusion proteins bound to glutathione agarose beads were incubated with recombinant myc-tagged GRIP_(PDZ4–7)_. Proteins complexes recovered from the beads were analyzed by immunoblotting. As shown in Figure 3B, we detected an association of GRIP_(PDZ4–7)_-myc with GST-Neto2_(CD)_, but not with GST-Neto2_(CDΔIDF)_. This observation confirms our earlier conclusion that the C-terminal tripeptide of Neto2 is necessary for its interaction with GRIP. For coimmunoprecipitation experiments, we expressed full length Neto2, Neto2 C-terminal truncation mutant (Neto2Δ7), or GRIP_(PDZ4–7)_ in COS-7 cells, and incubated the cell lysates with anti-Neto2 antibodies. We found that GRIP_(PDZ4–7)_ coimmunoprecipitated with anti-Neto2 antibodies only when coexpressed with Neto2, but not when it was expressed alone, or together with Neto2Δ7 (Figure 3C). This result shows that GRIP_(PDZ4–7)_ associates with full length Neto2 through a Neto2 C-terminal mediated interaction. To determine whether there is an interaction between native Neto2 and GRIP, we also performed coimmunoprecipitation experiments using cerebellar membrane fractions. Neto2 coimmunoprecipitated with the anti-GRIP antibody, but not with the negative control IgG, thus indicating that Neto2 and GRIP are indeed associated *in vivo* (Figure 4A). In summary, we have identified an interaction between Neto2 and the scaffolding protein GRIP. Furthermore, our studies indicate that this interaction is critically dependent on the C-terminal residues of Neto2.

To examine whether GRIP interacts specifically with Neto2, but not Neto1, as suggested by yeast two-hybrid studies, we expressed full length Neto1, and GRIP_(PDZ4–7)_ in COS-7 cells. In this system_,_ we found that GRIP_(PDZ4–7)_ did not coimmunoprecipitate with Neto1, thereby indicating a lack of interaction between the two proteins (Figure 3D). Additionally, to determine whether Neto1 functions in the same protein complex as GRIP through interactions mediated by additional proteins not present *in vitro*, we examined Neto1 and GRIP associations in the brain. In whole brain lysates, we did not observe coimmunoprecipitation of Neto1 with anti-GRIP antibodies (Figure 4B). On the other hand, coimmunoprecipitation of Neto2 was detected under the same conditions (Figure 4B). Based on these results, we conclude that GRIP does not interact with Neto1, and is not likely to be associated with it within the same protein complex *in vivo*.

### Neto2 forms a complex with GluK2 KARs and GRIP

**Figure 5:**
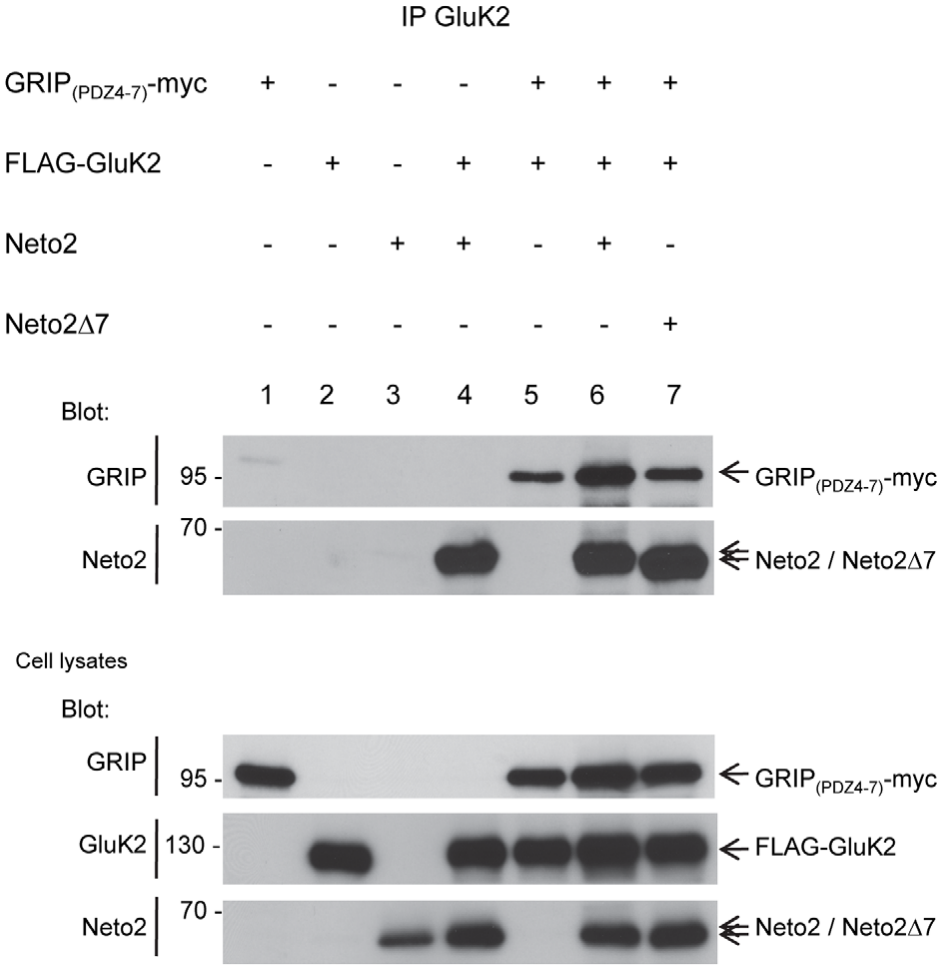
Neto2 increases the interaction between GRIP and GluK2. Immunoblot of immunoprecipitates from lysates of transfected COS-7 cells. The cDNAs used for transfection are shown above each lane. Neto2Δ7, Neto2 lacking the last seven C-terminal residues. Blot, antibody used for immunoblot analysis; IP, antibody used for immunoprecipitation.

We have previously shown that Neto2 interacts with the GluK2 subunit of KARs through its extracellular CUB domains [11], and here we showed that it binds to GRIP_(PDZ4–7)_ through its C-terminal tripeptide. Given that Neto2, GluK2, and GRIP can interact with each other, we examined whether coexpression of all three proteins leads to a competitive interaction or to the formation of a ternary complex. COS-7 cells were transfected with various combinations of expression plasmids FLAG-GluK2, Neto2, and GRIP_(PDZ4–7)_-myc. Cell lysates were immunoprecipitated with an anti-GluK2 antibody and proteins were analyzed by western blotting. As shown in Figure 5, the fraction of Neto2 that could be coimmunoprecipitated with GluK2 was not altered by co-expression with GRIP_(PDZ4–7)_ (lanes 4 and 6). Thus, GRIP_(PDZ4–7)_ does not compete or interfere with the Neto2:GluK2 interaction which occurs via the Neto2 ectodomain. On the other hand, when GluK2 and GRIP_(PDZ4–7)_ were coexpressed with Neto2, we observed a substantial increase in the amount of GRIP_(PDZ4–7)_ that coimmunoprecipitated with GluK2 (250%±41% of signal in the absence of Neto2, p<0.05; mean ± SD) (Figure 5, lanes 5 and 6). In contrast, coexpression of Neto2 lacking the last 7 C-terminal residues and which does not bind to GRIP_(PDZ4–7)_ had no effect on the amount of GRIP_(PDZ4–7)_ that coimmunoprecipitated with GluK2 (103%±26% of signal in the absence of Neto2, p<0.05; mean ± SD) (Figure 5, lanes 5 and 7). Based on these results, we conclude that Neto2, GRIP, and GluK2 can form a ternary protein complex.

## Discussion

In a recent study, we showed that Neto1, but not Neto2 plays a crucial role in regulating the abundance and channel kinetics of native KARs in the hippocampal CA3 region [11]. This result was unexpected given the fact that Neto2 was previously proposed as a main regulator of synaptic KARs [8]. In addition to the hippocampus, KARs are also abundantly expressed in the cerebellum, in particular, within the GCL [18], which appears to be devoid of Neto1 [13]. Thus, we asked whether Neto2, which is also abundant in cerebellar granule cells [13], regulates the synaptic localization of KARs in this brain region. In this report, we established that, in contrast to the hippocampus, loss of Neto2 resulted in ∼40% reduction in the level of GluK2 subunits at the cerebellar PSD. Given that strong GluK2-, and Neto2-immunoreactivity was concentrated in the granule cell layer, it is most likely that the loss of KARs occurred on the postsynaptic membrane of mossy fiber-granule cell synapses. Hence, we propose that in the cerebellum, Neto2 plays a major role in regulating the abundance of postsynaptic KARs.

In addition to a postsynaptic presence, KARs are also found presynaptically at granule cell axon (parallel fiber) terminals within the molecular cell layer of the cerebellar cortex. In the molecular layer, presynaptic KARs are involved in regulating the release of neurotransmitters [19]. Given that we have detected light to moderate Neto2 immunoreactivity throughout the molecular layer, it is possible that Neto2 may also be present at synapses within this region, such as those formed between parallel fiber and Purkinje neurons. It would, therefore, be interesting for future studies to explore any effect of Neto2 on KAR-mediated glutamate release at parallel fiber synapses.

While we have now shown that both Neto1 [11] and Neto2 are key players in the postsynaptic localization of native KARs, the two proteins appear to regulate receptors expressed in different neuronal populations. This region-specific regulation could be due in part to differences in the pattern of Neto1 and Neto2 expression. For instance, in the hippocampus, *Neto1* mRNA is particularly abundant in pyramidal cells of the CA3 region, whereas *Neto2* shows a relatively low but uniform distribution along the CA1-3 pyramidal layer [13], [17]. In the cerebellar cortex, Neto2 is strongly expressed in the GCL, while Neto1 appears to be absent from this region [13]. Other areas of high Neto1 and Neto2 expression in the brain include the amygdala, and the cerebral cortex ([17]; Allen Brain Atlas), both of which also express KARs [16], [20]. Whether Neto1 or Neto2 contribute to KAR abundance and function in these and other brain regions remain to be determined. The complementary, and in some cases, overlapping expression patterns for Neto1 and Neto2 throughout the brain are reminiscent of the differential distribution of members of the transmembrane AMPA receptor regulatory protein (TARP) family (γ-2, 3, 4, 5, 7,8). TARPs control AMPAR trafficking and gating, and their distinct regional distribution is thought to contribute to the synapse-specific regulation of their associated AMPARs [21]. For example, a spontaneous mutant of the cerebellar-enriched stargazin/γ2 subunit results in a selective loss of functional AMPARs in cerebellar granule neurons, but has no effect on receptors present in forebrain neurons, such as the CA1 pyramidal neurons [22], [23]. In contrast, loss of the γ-8 subunit, which is most highly expressed in the hippocampus but is absent from the cerebellum, severely reduced the levels of synaptic and extrasynaptic hippocampal AMPARs [24].

In addition to differences in neuronal expression, Neto1/2 might also regulate the synaptic localization of KARs depending on the subunit composition of the receptor. In a recent study by Copits and coworkers [9], coexpression of Neto2, and GluK1 in hippocampal culture neurons greatly enhanced the accumulation of GluK1-containing receptors to dendritic spines, and the colocalization of these receptors with the synaptic marker PSD95. Coexpression of Neto1, and GluK1, however, did not alter the GluK1 subcellular distribution, indicating that Neto2, but not Neto1, is responsible for the delivery/stability of synaptic GluK1-containing KARs. In contrast, we have previously shown that in the hippocampus, Neto1, but not Neto2, is crucial for the abundance of endogenous KARs at the PSD [11]. At MF-CA3 synapses, which show the highest expression of Neto1 in the hippocampus, postsynaptic KARs are predominantly GluK2/GluK5, GluK2/GluK4 heteromers [25], [26], [27], and do not contain GluK1 [18], [28]. Thus, the seemingly contradictory results from the hippocampus, and the culture neuron studies support the possibility that the preferential regulation of KARs by Neto1 or Neto2 could be, at least partially, attributed to the composition of the receptor being regulated. In the cerebellum, the subunit composition of KARs may also differ from that of the hippocampus. While both GluK2 and GluK5 subunits are strongly expressed (primarily in cerebellar granule cells) [18], GluK2 protein is ten times more abundant than GluK5 [29]. Given this difference in relative abundance, and the fact that granule cells do not appear to express other subunits, it is likely that the majority of cerebellar GluK2-containing KARs are GluK2 homomers. Neto2, may, therefore, be responsible for the synaptic localization of GluK2-KARs, in addition to that of GluK1-containing KARs, whereas Neto1 predominantly regulates GluK5-containing KARs.

Additional levels of KAR regulation might occur through the interaction of these receptors with different synaptic scaffolding proteins. GRIP is one such protein that has been shown to bind to GluK1 or GluK2 subunits in the brain [7]. Disrupting PDZ interactions between the cytoplasmic domain of KARs and its intracellular binding proteins, possibly GRIP, resulted in a significant reduction in KAR-mediated EPSCs at MF-CA3 synapses [7]. It has, therefore, been proposed that GRIP acts as an anchor that stabilizes KARs at the postsynaptic membrane, though this has not been demonstrated directly. In this study, we showed that Neto2 binds to GRIP directly through a C-terminal PDZ motif. Furthermore, coexpression of Neto2 with GRIP and GluK2 brings more GRIP into a complex containing GluK2. This is likely a result of either an increase in indirect interactions between GRIP and GluK2 through Neto2, or a stabilization of direct GRIP:GluK2 interactions by Neto2, or both. It is important to note, however, that GRIP expression in cerebellar GCL is relatively low, compared to, for example, the hippocampus [30]. Thus, in cerebellar granule cells, Neto2 may be responsible for bringing sufficient amounts of a limited number of GRIP molecules into the KAR protein complex in order to stabilize these receptors at the synapse. In hippocampal pyramidal cells, where GRIP expression is high, the role of Neto2 might not be essential. Generation of cell-specific Neto2 and GRIP knockout should address the intricacies and interdependencies of their expression in regulating synaptic KARs. It is possible that the interactions of Neto2, and possibly, Neto1, with various PDZ domain proteins, may add another regulatory layer to the trafficking and/or stabilization of KARs at various sites of action.

We have previously identified Neto1 as a key regulatory protein for the postsynaptic abundance of GluN2A-containing NMDARs and KARs in the hippocampus [11], [17]. Our discovery that Neto2 is also important for the synaptic accumulation of KARs, albeit at a different brain region, further strengthens the notion that Neto proteins are critical modulators of glutamate receptor function in the mammalian brain. Given the structural and sequence similarities between Neto1 and Neto2, future studies should explore whether Neto2 can also regulate the synaptic expression of NMDARs. Recently, a Neto-like protein has also been identified in Drosophila [31]. Disruption of *Drosophila* Neto in striated muscles of flies was found to severely compromise synaptic trafficking, and clustering of ionotropic glutamate receptors (iGluRs) at the PSD. Thus, similar to the loss of mammalian Neto1 and Neto2, Neto-deficiency in *Drosophila* significantly impaired the abundance of postsynaptic iGluRs. The similar function of mammalian Neto1, Neto2, and *Drosophila* Neto indicate these proteins are all evolutionarily conserved regulatory elements of glutamate receptors.

In addition to their role in regulating synaptic levels of KARs and NMDARs, the Neto proteins can also modify KAR channel properties. In heterologous cells, coexpression of Neto1 or Neto2 can significantly alter the rates of KAR entry and recovery from desensitization [8], [9], [10]. Similarly, at mossy fiber synapses, Neto1 is required not only for the synaptic expression of KARs [11], but also for generating the slow decay kinetics of KAR-mediated EPSCs [11], [12]. The dual role played by the Neto proteins on KARs raise the possibility that these two processes-receptor gating and localization- are not mutually exclusive. Meanwhile, *in vitro* studies indicate that the modulatory action of Netos on KAR kinetics may also vary according to the receptor subtype, akin to the subunit-dependent regulation of KAR synaptic targeting [9]. Thus, the differential regulation of KAR subtypes combined with distinct Neto1/2 expression patterns, and the potential association of Neto1/2 with diverse PDZ domain regulatory and scaffolding molecules constitute a possible mechanism for the synapse-specific regulation of KARs. How this multi-layered regulation impacts KAR-dependent neuronal signaling and transmission remains to be clarified.

## Materials and Methods

### Ethics statement

All animal care protocols and procedures were approved by the Toronto Center for Phenogenomics Animal Care Committee (Animal Use Protocol number: 12-10-0076-H).

### Mice

Mice were maintained at the Toronto Center for Phenogenomics. Generation of Neto2-null mice was described previously [11].

### Antibodies

Generation of antibodies to Neto1 and Neto2 is described elsewhere [11], [32]. Commercial antibodies: rabbit polyclonal antibodies to GluK2, GRID2 (Abcam), GluK5, GluA2/3, GRIP (Millipore), and normal rabbit IgG (Santa Cruz); mouse monoclonal antibodies to GluN1 (BD Biosciences), VAMP2 (Synaptic Systems), Homer (Abcam), Synaptophysin (Sigma), and GRIP (BD Transduction Laboratories).

### Two-hybrid interaction studies

The Neto2 cytoplasmic domain (Neto2_(CD)_, amino acids 368–525), and truncation mutant ΔIDF (Neto2_(CD-ΔIDF)_, amino acids 368–522) were amplified by PCR from mouse brain cDNA and fused to the yeast GAL4 DNA-binding domain in pDBLeu (Invitrogen). Mouse GRIP_(PDZ4–7)_ cDNA was obtained from the Toronto Centre for Applied Genomics facility, and subcloned in-frame with the GAL4 activation domain in pPC86 (Invitrogen). The controls used were mouse Neto1 (cytoplasmic domain) cloned into pDBLeu, and mouse PSD-95 and PICK1 cloned into pPC86. Vectors were transformed into MaV203 cells and interactions were scored by growth on triple dropout media (-Trp/-Leu/-His) and by testing for activation of the *lacZ* reporter gene.

### Mammalian expression constructs

Full length mouse Neto1 and Neto2 cDNA were subcloned into pcDNA3.1mycHisA(+) (Invitrogen) with a stop codon before the myc tag. GRIP_(PDZ4–7)_ cDNA was subcloned into pcDNA3.1mycHisA(+) in-frame with the myc epitope tag to generate C-terminal myc-tagged GRIP_(PDZ4–7)_. FLAG-GluK2 was a gift from Dr. Katherine Roche (National Institutes of Health, Bethesda, Maryland, USA).

### Cell culture and transfection

COS-7 cells (ATCC) were maintained at 37°C, 5% CO_2_ in Dulbecco's Modification of Eagle's Medium (DMEM) (Wisent) containing 10% fetal bovine serum (FBS) (Wisent), and were transfected with FuGene 6 (Roche). Forty-eight hours post-transfection, cells were lysed in 300 ul of RIPA buffer (50 mM Tris/HCl (pH 7.4), 150 mM NaCl, 1 mM EDTA, 1% Nonidet P-40, 0.5% deoxycholic acid (DOC), and 0.1% SDS) supplemented with Complete® Protease Inhibitor Cocktail tablets (Roche). Lysed cells were incubated on ice for 30 min, and centrifuged at 13,000× g for 15 min at 4°C. The protein concentration of the supernatant was determined using the detergent-compatible *DC* Protein Assay according to the manufacturer's protocol (Bio-Rad). Quantified samples were stored at −80°C, or used immediately for pull-down assays or coimmunoprecipitation experiments.

### Brain lysates and PSD

Tissue from wild-type and Neto2-null mice was homogenized in phosphate buffer saline, and centrifuged at 200× g for 5 min at 4°C. The pellet was resuspended in lysis buffer (50 mM Tris/HCl, (pH 7.4), 1 mM EDTA, and protease inhibitors), homogenized, and centrifuged at 10,000× g for 30 min at 4°C. The membrane pellet was homogenized in solubilization buffer (50 mM Tris/HCl (pH 7.4), 0.05 mM EDTA, 1% Triton X-100, 1% DOC, and protease inhibitors), rotated for 3 h at 4°C, and centrifuged at 10,000× g for 1 h at 4°C. The supernatant was used for immunoprecipitation. The PSD fraction was prepared as described previously [33] except that PSDs were extracted only once with 1% Triton X-100.

### Coimmunoprecipitation

COS-7 or brain lysates (1 mg protein) were incubated with antibodies overnight at 4°C, followed by incubation with 20–30 ul of GammaBind IgG beads (GE Healthcare) for 2 h at 4°C. Beads were washed extensively and bound proteins were eluted with SDS sample buffer (0.375 M Tris/HCl (pH 6.8), 60% (v/v) glycerol, 12% SDS, 0.06% bromophenol blue, and 0.6 M DTT) followed by SDS-PAGE/immunoblotting.

### In vitro binding assay (GST pull-down)

GST fusion protein constructs were generated in pGEX-4T-1 vector (GE Healthcare), and transformed into *E. coli* strain BL21. Bacterial cells were cultured for 18 h at 37°C in 2X YT medium containing 50 ug/ml ampicillin. This culture was subsequently inoculated at 1∶100 dilution into fresh 2X YT medium containing ampicillin, and grown at 37°C until an OD_600_ of 0.5–0.8 was reached. Protein expression was induced with 1 mM isopropylthio-ß-galactosidase (IPTG) for 3 h at 30°C. Cells were centrifuged at 3400× g for 10 min at 4°C, and the resulting pellet was lysed with B-Per Bacterial Protein Extraction Reagent according to manufacturer's protocol (Pierce). Fusion proteins were purified on glutathione agarose beads (Sigma), and incubated with COS-7 cell lysates overexpressing GRIP_(PDZ4–7)_-myc protein overnight at 4°C. Beads were washed four times with RIPA buffer minus SDS and DOC (50 mM Tris/HCl (pH 7.4), 150 mM NaCl, 1 mM EDTA, 1% Nonidet P-40) supplemented with Complete® Protease Inhibitor Cocktail tablets (Roche). Bound proteins were eluted with SDS sample buffer followed by SDS-PAGE gel and immunoblotting.

### Immunohistochemistry

Brains were dissected out after intracardiac perfusion, cryo-protected in 30% sucrose before embedding in OCT, and sectioned at 50 µm-thickness. Slices were fixed with 4% PFA/PBS for 1 minute on ice, and blocked with 10% goat serum, 0.3% Triton X-100 in PBS at room temperature for 1 h. Slices were incubated with primary antibodies overnight at 4°C, and secondary antibodies for 1 h at room temperature, followed by 24 h curing at room temperature with Prolong Gold antifade (Invitrogen). Images of slices from the same glass slide were acquired with fixed exposure settings using a Zeiss LSM 510 confocal microscope.
